# The Role of Endothelial Senescence in the Pathogenesis of Diabetic Retinopathy

**DOI:** 10.3390/ijms26115211

**Published:** 2025-05-29

**Authors:** Manav Gandhi, Shahzaib Haider, Helena Zin Ying Chang, Andrius Kazlauskas

**Affiliations:** 1 Department of Physiology and Biophysics, University of Illinois Chicago, Chicago, IL 60612, USA; mgandh23@uic.edu (M.G.); hchang77@uic.edu (H.Z.Y.C.); 2 Chicago School of Medicine, Rosalind Franklin University of Medicine and Science, North Chicago, IL 60064, USA; zaib.haider09@gmail.com; 3 Department of Ophthalmology and Visual Sciences, University of Illinois Chicago, Chicago, IL 60612, USA

**Keywords:** senescence, diabetic retinopathy, resilience to diabetic retinopathy

## Abstract

Diabetic retinopathy (DR) is the most common microvascular complication of diabetes mellitus (DM). Key drivers of DR include mitochondrial dysfunction, oxidative stress, and chronic inflammation, which lead to premature senescence of cells within the retinal vasculature. Senolytics improve outcomes in both animal models and in patients with severe forms of DR. In this review, we discuss (i) the role of endothelial senescence in each stage of DR pathogenesis, (ii) methods for detecting senescence in cultured endothelial cells and retinal vessels, and (iii) potential mechanistic explanations for how cells within retinal vessels resist DM-driven senescence.

## 1. Senescence Versus Aging

Aging is a complex biological process marked by the gradual decline in physiological functions across organ systems, ultimately leading to increased susceptibility to disease and mortality. This decline is driven by a range of molecular and cellular alterations, including genomic instability, telomere attrition, epigenetic dysregulation, mitochondrial dysfunction, and impaired proteostasis. These hallmarks collectively contribute to diminished regenerative capacity, tissue degeneration, and systemic inflammation that define the aging phenotype [1]. New insights suggest that cellular senescence—a state of stable growth arrest—is not merely a byproduct of aging but a causal and dynamic contributor to the process [2,3].

Senescence is traditionally understood as a protective program triggered in response to stressors such as DNA damage, oncogene activation, oxidative stress, or telomere shortening. It is enforced through activation of the p53/p21 and p16^INK4a/Rb^ tumor suppressor pathways, which irreversibly halt cell division. Although originally conceptualized as a static, terminal state that protects against cancer by halting the proliferation of damaged cells, recent findings indicate that senescence is a progressive and evolving process. Senescent cells undergo chromatin remodeling and metabolic reprogramming and develop a distinct secretory profile known as the senescence-associated secretory phenotype (SASP). The SASP composition includes pro-inflammatory cytokines, chemokines, growth factors, and matrix-modifying enzymes that can profoundly alter the surrounding tissue microenvironment [2,3]. The level of some SASPs is elevated in the vitreous of patients with diabetic retinopathy (DR) [4,5], which implicates senescence in DR.

Attempts to better understand senescence and its impact on pathogenesis have included its stratification into acute and chronic forms of senescence. Acute senescence occurs rapidly and serves beneficial roles in development, wound healing, and limiting fibrosis, where senescent cells are effectively cleared by the immune system. In contrast, chronic senescence arises gradually in response to persistent stressors, is less efficiently cleared—especially with age-related immune decline—and contributes to the accumulation of dysfunctional cells in tissues. These chronically senescent cells can promote tissue degeneration through paracrine signaling (via SASPs) that spreads senescence to neighboring cells, degrades the extracellular matrix, and disrupts stem cell niches, leading to impaired tissue maintenance and repair.

The relationship between aging and senescence is thus deeply intertwined and bidirectional. While senescence prevents tumorigenesis and facilitates tissue remodeling, its prolonged presence accelerates aging through inflammation, structural damage, and stem cell exhaustion. This duality exemplifies the concept of antagonistic pleiotropy, where a process beneficial early in life becomes detrimental with age. Therapeutic interventions aimed at selectively eliminating or reprogramming senescent cells—such as senolytics or SASP-modulating agents—are increasingly being investigated as strategies to mitigate age-related diseases and extend healthspan [1,2,3]. As outlined below, our appreciation that diabetes (DM) accelerates senescence of the retinal vasculature and that senescence can either exacerbate or mitigate pathogenesis is guiding the use of senescence-directed therapeutics for DR.

## 2. Biological Versus Chronological Age

Recent studies provide strong evidence that the retinal age gap (RAG)—the difference between biological and chronological age derived from retinal imaging—serves as a novel and predictive biomarker for aging-related diseases, including DR.

Chronological age (CA) is simply the number of years a person has lived, whereas biological age (BA) reflects the physiological state of the body, capturing how well or poorly one’s tissues and organs are aging. While CA is a fixed and straightforward measure, BA is dynamic and varies based on genetics, lifestyle, and disease burden. BA can be estimated through various methods, such as DNA methylation (epigenetic clocks), telomere length, and retinal fundus photographs.

Several studies have demonstrated that a higher RAG correlates with a higher risk of DR. For instance, among individuals with DM who have not developed DR, each year of RAG raised the risk of developing DR by 7% [6]. Similarly, RAG increases progressively with the severity of DR—from 0.6 years in patients with DM without DR to over 8.5 years in those with proliferative DR [7]. These observations beg the question of whether the accumulation of senescent cells within the retinal vasculature increases the RAG.

## 3. Evidence That the Neural Retina Ages

A growing body of evidence confirms that the retina, like many other neural tissues, undergoes significant structural, functional, and molecular changes with age. In humans, these changes manifest across nearly every retinal layer and cell type. Structural alterations include thinning of the retinal nerve fiber layer (RNFL), ganglion cell layer (GCL), inner nuclear layer (INL), and outer nuclear layer (ONL), as well as disruption of the retinal pigment epithelium (RPE) and Bruch’s membrane [8,9]. Rod photoreceptors are particularly vulnerable, undergoing substantial cell loss in both the central and peripheral retina, which contributes to the decline in scotopic (low-light) vision commonly observed in older adults [8].

Functionally, these anatomical changes translate into measurable declines in visual performance. Older adults frequently experience reductions in visual acuity, contrast sensitivity, dark adaptation, and color discrimination [8,9]. Electroretinography (ERG) reveals age-related declines in both a- and b-wave amplitudes—reflecting photoreceptor and bipolar cell dysfunction—and increased implicit times, indicating slower neural processing [9]. Multifocal ERG studies further show preferential loss in central retinal function, particularly affecting cone-rich areas. Interestingly, despite these functional impairments, the total number of certain retinal neurons remains relatively stable with age, suggesting that synaptic disconnection and altered signaling rather than cell loss may be primary contributors to functional decline.

At the cellular and synaptic level, the aging retina shows evidence of significant plasticity. In aged human retinas, bipolar and horizontal cells undergo dendritic remodeling, often extending their processes into inappropriate layers such as the ONL, likely in response to upstream photoreceptor degeneration [10]. These alterations are often accompanied by glial activation, including astrocyte hypertrophy and microglial proliferation, reflecting the retina’s attempt to maintain homeostasis in the face of chronic stress.

Mouse models reveal similar trends. In C57BL/6J mice aged 2 to 32 months, progressive retinal thinning—especially of the ONL—is observed alongside declines in rod- and cone-mediated ERG responses [11,12]. These findings were extended by demonstrating that aging increases the retina’s susceptibility to mild stressors such as elevated intraocular pressure (IOP) [13]. Their data show that aged retinas respond to IOP with robust activation of inflammatory and senescence-related genes, including p16^Ink4a^ and SASP factors like IL-6. Furthermore, repeated stress exposures in young mice accelerated both transcriptional and DNA methylation aging, closely mimicking the molecular landscape of naturally aged retinas. These findings were supported by chromatin accessibility data, showing that enhancers associated with inflammatory and senescence pathways become more active with age and stress.

These structural, functional, and molecular shifts illustrate that the retina is an aging-sensitive tissue shaped by cumulative physiological and environmental insults. In light of the intimate physical and functional relationship between the neural and vascular components of the retina, it is likely that endothelial senescence and the resulting vascular dysfunction drive age-related changes in the neural retina [8,13]. The recent appreciation that aging changes gene expression in photoreceptors to a much greater extent than in vascular cells [14] suggests that age-related neural dysfunction is likely to precede and even contribute to vascular dysfunction.

## 4. The Effect of Senescence Is Dependent on the Stage of DR Pathogenesis

The composition of the SASP, which changes as DR progresses, is a key determinant of how senescent endothelial cells influence pathogenesis (Table 1) [15]. Once resilience to DR (RDR) deteriorates and the retinal vasculature begins to accumulate senescent cells, SASPs are predominantly pro-inflammatory, marked by elevated IL-1β, IL-6, TNF-α, ICAM-1, VEGF, and matrix metalloproteinases, which drive inflammation and increase vascular permeability [15]. In the advanced, vision-threatening stages of DR, particularly during the regression of pathological neovascularization in proliferative DR, the SASP composition can shift. At this point, senescent endothelial cells begin to secrete cytokines such as IL-8 and CXCL1 that recruit neutrophils and mononuclear phagocytes, leading to immune-mediated clearance of senescent endothelial cells and regression of pathological angiogenesis [4,16]. Thus, the net effect of senescent endothelial cells in the pathogenesis of DR is shaped by the composition and timing of SASP expression. These discoveries have catalyzed pre-clinical and clinical studies testing the benefit of senolytics for patients with advanced forms of DR. UBX1325 (foselutoclax) was effective in animal models of retinopathy arising from vascular dysfunction and safe in patients with diabetic macular edema [17,18].

Building on the favorable safety profile observed in recent Phase 1 clinical trials [17,18], UBX1325 emerges as a promising senolytic therapy for diabetic macular edema. It Is a small molecule that antagonizes Bcl-XL, a member of pro-survival pathways that are up-regulated in senescent cells, allowing them to resist apoptosis despite DNA damage and oxidative stress [19,20,21,22,23,24,25,26]. By antagonizing Bcl-XL, UBX1325 selectively eliminates senescent endothelial cells while sparing healthy ones. However, Bcl-XL also plays an essential role in maintaining retinal neuronal survival [27,28]. Thus, while early clinical data are en-couraging, ongoing evaluation is needed to ensure that targeting Bcl-XL does not inad-vertently impair neuronal function in the retina.

## 5. DM Exacerbates Processes That Promote the Senescence of Endothelial Cells Within Retinal Vessels, Thereby Accelerating the Senescence of the Retinal Vasculature

**Figure 1:**
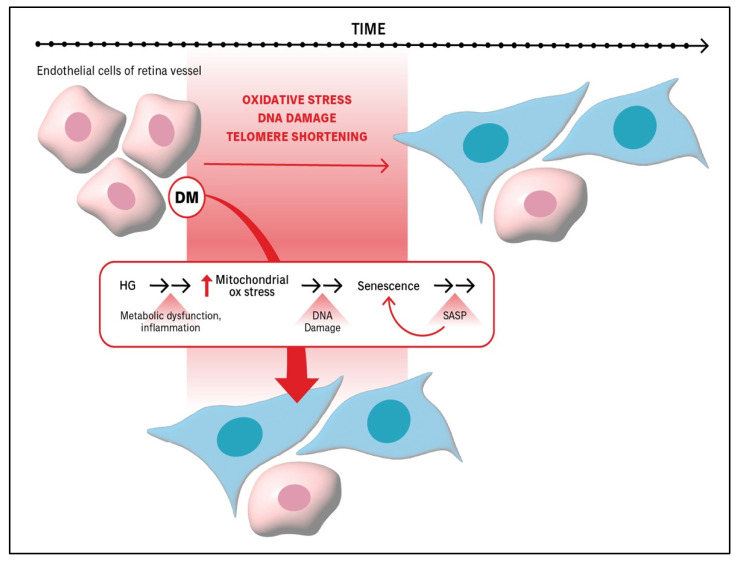
**Diabetes exacerbates the molecular and biochemical drivers of senescence.** Oxidative stress, DNA damage, and telomere shortening are some of the drivers of senescence. In patients with diabetes (DM), chronic hyperglycemia (HG) intensifies these processes and thereby accelerates the onset of senescence. The senescence-promoting processes that HG exacerbates include metabolic dysfunction and inflammation, which elevates mitochondrial oxidative stress and results in DNA damage, which triggers senescence. The senescence-associated secretory phenotype (SASP) is an increase in the production of pro-inflammatory cytokines, chemokines, growth factors, and matrix-modifying enzymes. The increased level of such agents is not only diagnostic of senescence but also enforces and propagates senescence to neighboring cells. oxidative (ox).

DM promotes processes that induce senescence of the endothelium within retina vessels (Figure 1) [15,29]. Hyperglycemia, the central metabolic disturbance in patients with DM, promotes endothelial cell senescence through sustained oxidative stress, inflammation, and nitric oxide (NO) deficiency. Chronically elevated glucose levels activate multiple metabolic pathways—such as the polyol, hexosamine, protein kinase C (PKC), and advanced glycation end product (AGE) pathways—that converge on increased production of reactive oxygen species (ROS). These ROSs cause DNA damage and activate the DNA damage response, driving cells into a state of irreversible cell cycle arrest via the p53/p21 and p16INK4A/RB pathways. Mitochondrial dysfunction (see the detailed description below) and reduced antioxidant capacity exacerbate ROS accumulation, further promoting cellular damage. At the same time, hyperglycemia suppresses endothelial nitric oxide synthase (eNOS) activity and depletes NO, which normally acts as an anti-senescent, vasoprotective molecule. The combined effects of DNA damage, mitochondrial stress, and impaired NO signaling establish a cellular environment that favors premature senescence.

Recent studies have identified the cGAS-STING signaling cascade as an additional driver of endothelial senescence in the context of DR [30,31]. In diabetic retinal tissues from both human patients and mouse models, STING expression is upregulated and localized to endothelial cells in neovascular membranes. This pathway, normally involved in innate immune responses to cytoplasmic DNA, becomes aberrantly activated by mitochondrial and nuclear DNA damage in hyperglycemic environments. Activation of STING leads to phosphorylation of TBK1 and IRF3, inducing the production of type I interferons (IFNs), which promote endothelial cell senescence [30]. Additionally, STING activation triggers NF-κB-mediated inflammation, increasing levels of cytokines like TNF-α, IL-1β, and IL-6, which are components of SASPs that further reinforce both senescence and vascular damage.

## 6. Approaches to Detect Senescence Within Retinal Vessels and Cultured Cells

Detection of senescent cells requires multiple complementary approaches, as no single biomarker is universally definitive. The various approaches can be broadly grouped as morphological, histochemical, and enzymatic assays, detection of cell cycle regulators, chromatin and epigenetic alterations, SASP analysis, and omics approaches. Table 2 provides a summary of these approaches that were used to detect senescence in retinal vessels and cultured cells.

One of the most common methods is the senescence-associated β-galactosidase (SA β-Gal) assay. β-galactosidase is a lysosomal enzyme that is active at pH 4 in homeostasis, but when cells undergo senescence, there is an accumulation of pH 6 active β-galactosidase. This assay is not universal for senescence; certain immune cells can also show β-galactosidase activity [32]. Ki-67 staining exclusively marks proliferating cells, and hence a negative marker of senescent cells. Furthermore, senescent cells have distinct morphological alterations, such as flattened, enlarged, and irregularly shaped cells, which can be easily visualized under brightfield microscopy.

Cell cycle arrest is a classic feature of senescent cells and is usually mediated by cyclin-dependent kinase inhibitors like p16^INK4A^ and p21^CIP1/WAF1^, which prevent phosphorylation of retinoblastoma (p-Rb) protein and thereby result in induce stable G1 arrest. Changes in the expression of p16INK4A and p21CIP1/WAF1 are typically determined via Western blot or qPCR. DNA damage is a key trigger for cell cycle arrest and coincides with the induction of senescence. This causes the accumulation of p53, which further increases the expression of p21. The loss of Lamin B1, a nuclear protein, compromises nuclear envelope integrity and promotes the entry of DNA-damaging insults into the nucleus. Loss of Lamin B1 can be detected using qPCR, Western blot, or immunofluorescence. Such dramatic nuclear changes are also associated with the formation of senescence-associated heterochromatic foci, which are regions of chromatin enriched in modification like H3K9me3, a characteristic of oncogene-induced senescence.

SASP production is another defining feature of senescence that contributes to inflammation and includes soluble factors like interleukins, chemokines, growth factors, inflammatory factors, insoluble ECM factors, shed receptors or ligands, and nonprotein soluble factors. A comprehensive list of various SASPs is discussed herein [33]. SASPs can be measured using ELISA, mass spectrometry, or RNA sequencing, and the profile of SASP depends on factors like stressors, cell types, and tissue environments. Finally, in vivo studies have greatly benefited from omics-based techniques like bulk RNA-seq or single-cell RNA-seq which provides unprecedented resolution for detecting gene expression profiles of senescent cell population.

The application of additional experimental approaches, such as p16 reporter mice that allow real-time tracking of senescent cells, is likely to further advance our ability to observe and understand the role of senescence in DR pathogenesis [34,35].

**Table 2:** Approaches to detect senescence in retinal vessels and cultured retinal endothelial cells.

Disease State	Experimental System	Technique	Readouts	References
DR	Retinal Explant	ELISA	↑ HMGB1 ↑ IL-1β	[30]
	mRECs	Flow Cytometry	Cells positive for CellEvent Senescence Green Flow Cytometry Assay	
		Immunocytochemistry	↑ β-galactosidase activity	
		Cell Proliferation	Edu assay	
	HRECs	Immunocytochemistry	↑ β-galactosidase activity	
		Cell Proliferation	Edu assay	
	Murine RV	PCR	↑ p16 ↑ p21 ↑ Igfbp3 ↑ p53	
	Freshly Isolated Murine RV	SA-β-Gal Assay	↑ β-galactosidase activity	
DR and neovascular retinal diseases	HMRECs	PCR	↑ Cdkn2a (Ink4a) ↑ Cdkn1a	[36]
	OIR P17 Retina	PCR	Concomitant peaks of senescence (↑ Cdkn2a (Ink4a), Cdkn1a, Serpine1, Vegfa) and maximal pathological angiogenesis	
		SA-β-Gal Assay	Maximal stained cells in inner retina where new vessels form	
		Western Blot	p16INK4a PAI1 (product of Serpine1 gene)	
		DropSeq	GSVA showed ↑ senescence transcripts in clusters of endothelial cells, pericytes, astrocytes, Müller glia	
		10X Single-Cell RNA Seq	Supervised clustering for senescence showed enrichment in endothelial cells	
DR	HRECs	SA-β-Gal Assay	↑ β-galactosidase activity	[37]
		Western Blot	↑ p21	
		Flow Cytometry	G0/G1cell cycle arrest	
DR	HMRECs	PCR	↑ p53 ↑ p16 ↑ p21 ↑ IL-1β ↑ IGFBP7	[38]
		Western Blot	↑ p53 ↑ IL-1β ↑ IGFBP7 ↑ ICAM-1 ↑ MCP-1 ↑ MMP9	
		SA-β-Gal Assay	↑ β-galactosidase activity	
		Immunofluorescence	↑ p53	
	db/db mouse retina	Single-Cell RNA Seq	Enrichment of senescence and p53 pathways in KEGG pathway analysis for endothelial cluster	
			p53 enrichment only in endothelial cluster	
Prolonged DM and DR	HMRECs	CASY Cell counter	↓ growth	[39]
			↑ cell diameter	
			↑ cell area	
			↓ population doublings	
			↓ Hayflick limit	
		SA-β-Gal Assay	↑ β-galactosidase activity	
		Western Blot	↓ pRb ↓ HMGB2 ↓ Sirt1 ↑ p53 p21, p16 (although inconsistent change)	
	db/db mouse retina	Histology	Costaining of β-galactosidase activity and Isolectin B4	
	Akimba mouse	Single-Cell RNA Seq	Enrichment of SASP and senescence genes in ECs	
Retinopathy	OIR P17 Retina	Single-Cell RNA Seq	Enrichment of senescence and SASP in endothelial cell cluster	[16]
			GSVA indicating enrichment of senescence transcripts	
		Confocal	Colocalization of Isolectin B4 and promyelocytic leukemia (PML) protein for senescent endothelial cells	
			Colocalization NG2 and promyelocytic leukemia protein for pericytes	
			RAS colocalization with Isolectin B4+ endothelial cells in preretinal NV	
			Colocalization of Isolectin B4 and high pERK1/2	
	OIR P19 Retina	SA-β-Gal Assay	β-galactosidase activity	
		PCR	↑ p21 ↑ Serpine1 ↑ IL-1β ↑ p53 ↑ Vegfa ↑ Tnfα ↑ Tgfβ	
NA	HRECs	SA-β-Gal Assay	↑ β-galactosidase activity	[40]
		PCR	↑ p53	
		Population doubling time	↑	
		DCF Assay	↑ intracellular oxidative stress	
NA	HRECs	SA-β-Gal Assay	↑ β-galactosidase activity	[41]
		PCR	↑ CDKN1A (p21 CIP1) ↑ IL6	
Chronic DM and implication in DR	HRECs	SA-β-Gal Assay	↑ β-galactosidase activity	[42]
		TRAP PCR Assay	↓ Telomerase activity	
		PCR	↑ p53 ↑ p21 ↓ SIRT1 (regulator of senescence)	
		Western Blot	↑ p53 ↑ p21 ↓ SIRT1 (regulator of senescence)	
NA	HRECs	TRAP PCR Assay	↓ Telomerase activity	[43]
		PCR	↑ PAI-1 ↑ TERF2 ↓ TERT ↓ SIRT1	
		SA-β-Gal Assay	↑ β-galactosidase activity	
		Western Blot	↑ PAI-1 ↓ SIRT1 (regulator of senescence)	
DR	HMRECs	Western Blot	↑ p21 ↓ SIRT3 (regulator of senescence)	[44]
		SA-β-Gal Assay	↑ β-galactosidase activity	
DME	HMRECs	Ionizing radiation	Actin filament organization showing impaired monolayer formation	[17]
		Alamar Blue Assay	↓	
		Standard microscopy	↓ cell density	
		SA-β-Gal Assay	↑ β-galactosidase activity	
		Fluorescence microscopy	↑ PML bodies	
		Western Blot	↑ p16 ↑ p21 ↑ p53	
		PCR	↑ CDKN1A ↑ CDKN2A/INK4a ↑ SERPINE1 ↑ IL6 ↑ IL8 ↑ TNF ↑ERN1	
		Immunoblot	↑ γH2AX ↑ PAI-1 (SERPINE1)	
	Human Retina	Immunofluorescence	↑ p16INK4A	
			Colocalization of PAI-1 and COL4-positive ECs	
	STZ Retina	Single-Cell RNA Seq	Enrichment of senescence signature in ECs	
NA	HRECs	SA-β-Gal Assay	↑ β-galactosidase activity	[45]
		Western Blot	↑ p16Ink4a ↑ p21Waf1 ↓ SIRT1 (regulator of senescence)	

DM—diabetes mellitus, DR—diabetic retinopathy, DME—diabetic macular edema, HRECs/HMRECs—primary human retinal/microvascular endothelial cells, mRECs—mouse retinal endothelial cells, OIR—oxygen-induced retinopathy mouse model, STZ—Streptozotocin-induced type 1 diabetes mouse model. The up- and down-pointing arrows indicate the direction of the change in expression.

## 7. The Role of Mitochondria in Senescence

Mitochondria play a crucial role in cellular senescence by regulating energy metabolism and ROS production [46]. Accumulation of dysfunctional mitochondria results in increased oxidative stress and metabolic imbalances, processes that drive cells into senescence.

Mitophagy, a selective autophagy process targeting damaged mitochondria, declines with age and in senescent cells [47]. Reduced mitophagy leads to the persistence of dysfunctional mitochondria, exacerbating cellular stress and reinforcing the senescent state. Studies suggest that interventions enhancing mitophagy, such as caloric restriction or pharmacological agents like Urolithin A, may counteract age-related mitochondrial dysfunction and mitigate senescence [48,49,50].

Moreover, mitochondrial dysfunction influences senescence through metabolic shifts [51]. Cells undergoing mitochondrial dysfunction-associated senescence exhibit distinct metabolic alterations, including changes in the NAD+/NADH ratio, AMPK activation, and p53 pathway modulation. These changes arrest cell proliferation and modify the composition of SASP, leading to differential effects on tissue homeostasis and aging.

Mitochondrial dysfunction within the retina is associated with early DR in both patients and experimental animals, and this damage is caused by hyperglycemia-driven elevation of mitochondrial oxidative stress [29,52,53,54,55]. There is an increasing appreciation that such changes occur in the endothelium of retinal blood vessels and contribute to the pathogenesis of DR [30,56].

## 8. The Role of Mitochondria in Cell Fate Decisions

Mitochondria serve as critical regulators of cell fate by modulating the balance between apoptosis and senescence [47,57]. Apoptosis, or programmed cell death, is a tightly controlled process that eliminates damaged or dysfunctional cells to maintain tissue integrity. Mitochondria play a central role in apoptosis by releasing pro-apoptotic factors such as cytochrome c, which activates the caspase cascade leading to cell death. When cellular stress is severe, mitochondria initiate this pathway to prevent the propagation of damaged cells that could contribute to disease. However, when stress is moderate or persistent but not lethal, cells may instead enter a senescent state, halting proliferation while remaining metabolically active.

While apoptosis eliminates damaged cells, senescence allows them to persist, sometimes leading to detrimental consequences at the tissue and organismal levels. Thus, targeting mitochondrial pathways involved in senescence and apoptosis offers promising therapeutic avenues for mitigating age-related dysfunction [58]. While this topic is applicable to many cell types, several review articles focus on the influence of the mitochondrial on cell fate decisions in endothelial cells and endothelial cell precursors [59,60,61].

## 9. Genes That Initiate and Commit Cells to Senescence

The complex contribution of endothelial cell senescence to the pathogenesis of DR (Table 1) warrants investigation of the underlying mechanism. As described below, some of the molecular mediators of senescence have been identified using model systems. A summary of these discoveries is provided to guide the identification of genes responsible for inducing endothelial cell senescence in response to prolonged hyperglycemia.

Cellular senescence is triggered and maintained by an intricate interplay of signaling pathways, transcription factors, and chromatin modifiers. The canonical tumor suppressor pathways involving p53/p21^CIP1^ and p16^INK4a/Rb^ are fundamental in initiating cell cycle arrest in response to DNA damage, oncogene activation, or oxidative stress. However, these well-known checkpoints are modulated by a broader network of gene regulators that either reinforce or modulate the senescence program.

The senescence restriction point (SeRP) is a chromatin-based commitment mechanism that integrates the intensity and duration of oncogenic signals [62]. Sustained activation of the RAS–MAPK–ERK pathway initiates chromatin remodeling via transcription factors such as ETV4 and RUNX1, which act as central integrators of oncogenic stress. Once the SeRP is passed, chromatin regions—especially nucleolar-associated domains—become irreversibly accessible, locking in the senescent fate even after the initial signal dissipates.

The AP-1 transcription factor complex (including members like c-JUN and FOS) also plays a pioneering role in establishing the enhancer landscape that dictates senescence competence. AP-1 primes enhancer elements early in the senescence program, defining a hierarchy of transcriptional regulators that orchestrate senescence-specific gene expression [63]. This enhancer remodeling leads to dynamic but structured activation of SASP and cell cycle arrest modules, distinguishing senescence from reversible cell cycle exits like quiescence.

In parallel, NF-κB (particularly the p65 subunit) functions as a master regulator of SASP production. NF-κB binds to senescent chromatin more extensively than even p53 or Rb and is critical for both SASP gene expression and immune-mediated clearance of senescent cells [64]. Inhibition of NF-κB leads to SASP suppression, escape from immune surveillance, and resistance to senescence-inducing chemotherapy.

While classical models emphasize p16^INK4a^, p21^CIP1^, and p15^INK4b^ as direct enforcers of senescence via inhibition of cyclin-dependent kinases, newer findings show that other mechanisms can converge on the Rb pathway. STAT5A-induced senescence involved downregulating Myc—a driver of CDK4 expression—thereby indirectly activating Rb without involving p16 or p16^INK4A^ and p21^CIP1/WAF1^ [65]. Restoration of Myc or CDK4 bypassed senescence even in p53-deficient cells, underscoring the modularity and adaptability of senescence control mechanisms.

The balance between transcriptional activators and repressors also fine-tunes the commitment to senescence. Ets family proteins activate the p16^INK4a^ promoter to enforce senescence, whereas Id proteins suppress it, counteracting Ets activity [66]. The relative expression of these factors influences whether a cell commits to senescence, suggesting a bistable regulatory system governing senescence entry.

Senescence can be reinforced through paracrine signaling. IL-1-mediated inflammasome activation in senescent cells amplifies SASP and triggers p21^CIP1^ and p16^INK4b^ expression in neighboring cells, propagating senescence in a non-cell-autonomous fashion [67]. This paracrine senescence relies on TGF-β, VEGF, and chemokines like CCL2/CCL20, placing the SASP not only downstream but upstream of senescence propagation.

**Figure 2:**
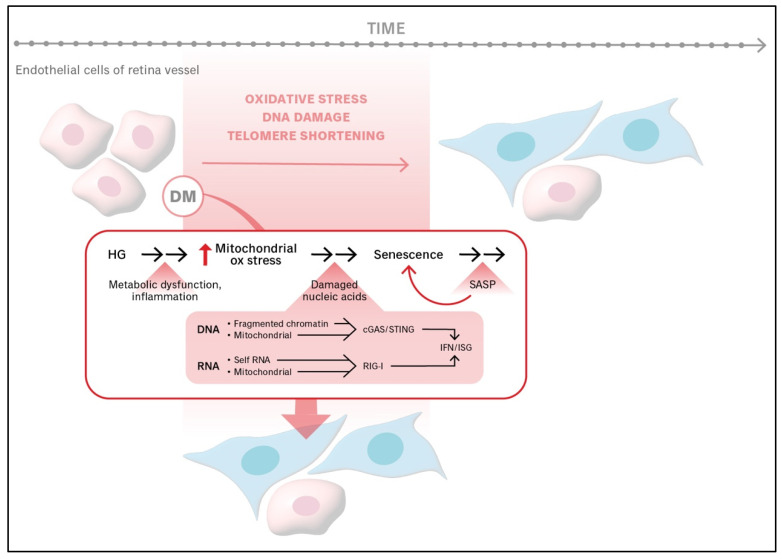
**Molecular mediators of senescence in endothelial cells.** Mitochondria oxidative stress damages both DNA and RNA, which act via the cGAS/STING and RIG-I pathways, respectively, to increase the expression of interferon (IFN) and interferon-stimulated genes (ISG) [30,31,56]. These changes promote senescence, at least in part, by inducing the expression of senescence-associated secretory phenotype (SASP) [70]. diabetes mellitus (DM); hyperglycemia (HG); oxidative (ox).

Recently published papers showed that nucleic acids mediate senescence in endothelial cells (Figure 2). Insults such as chronic hyperglycemia, damage both nuclear and mitochondrial DNA, which activates the cGAS-STING signaling cascade and thereby increases the level of interferon [30,31,68]. Similarly, RNA, including mitochondrial RNA that has escaped from damaged mitochondria, acts via the RNA sensor retinoic acid-inducible gene I (RIG-I) to promote the expression of interferon-stimulated genes (ISG)s [56,69].

## 10. Resilience to DR (RDR)

**Figure 3:**
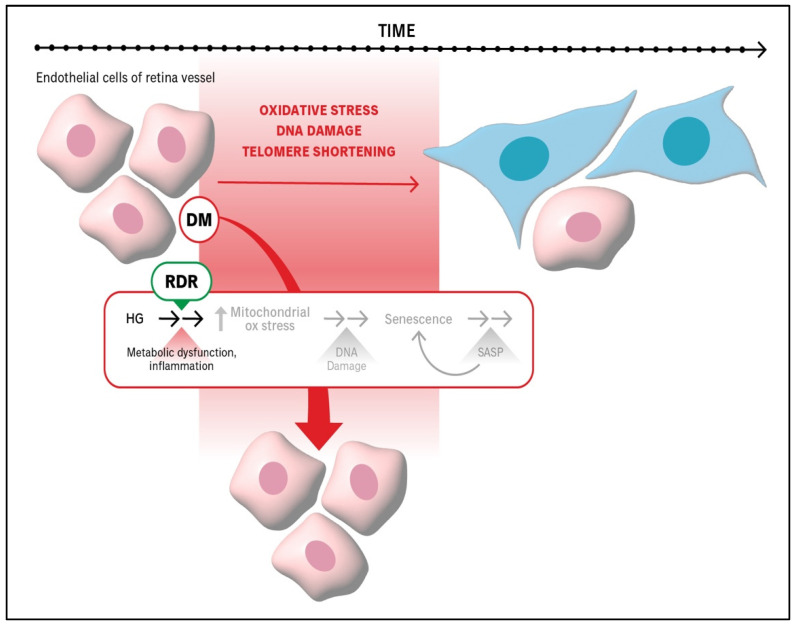
**RDR prevents hyperglycemia-driven processes that accelerate senescence.** Resilience to DR (RDR) protects retinal endothelial cells from hyperglycemia (HG)-induced senescence. The underlying mechanism involves enhanced mitochondrial flux; a matched increase in mitochondrial biogenesis and disposal (via mitophagy) with no net change in the mitochondrial mass [29]. diabetes mellitus (DM).

The concept of RDR refers to the retina’s innate ability to remain healthy in the face of chronic hyperglycemia [29] (Figure 3). The underlying mechanism of RDR has been investigated with participants of the Joslin 50-Year Medalist Study: individuals with type 1 diabetes (T1D) who did not develop DR for over 50 years, independent of tight glycemic control [71]. This delay in DR onset, despite chronic hyperglycemia, suggests the presence of protective mechanisms intrinsic to the retina. One such factor identified in these resilient individuals is retinol-binding protein 3 (RBP3), which reduces glucose uptake in photoreceptors and attenuates inflammatory signaling, thereby limiting hyperglycemia-induced retinal damage.

Studies with animal and cell culture models of RDR have indicated that mitochondrial adaptation to hyperglycemia is a key mechanism underlying RDR (Figure 3) [72,73,74]. Endothelial cells respond to persistent hyperglycemia by increasing mitochondrial flux, which involves a balanced increase in mitophagy and mitochondrial biogenesis such that there is no net change in mitochondrial mass. These adaptations prevent the accumulation of damaged mitochondria and minimize oxidative stress, thereby preventing senescence.

Deterioration of RDR is a newly appreciated component of DR pathogenesis. This breakdown in resilience represents a critical transition point in disease development. In both patients and experimental animals, the loss of RDR marks the shift from a damage-resistant to a damage-prone retina [29]. This transition is associated with the failure of mitochondrial homeostasis, particularly a mismatch between mitophagy and biogenesis, resulting in the accumulation of dysfunctional mitochondria. DNA and RNA can leak out of damaged mitochondria, into the cytoplasm and can trigger senescence via the cGAS/STING and RIG-1 pathways. Therapeutically, this presents an opportunity: strategies that preserve or restore RDR could indefinitely delay the onset or progression of DR. As such, RDR-based therapies represent a promising frontier in DR research and intervention.

## 11. Conclusions

RDR antagonizes processes that promote the senescence of retinal endothelial cells. Suppression of endothelial senescence maintains vascular integrity and delays the onset of damage to the retina, which is diagnosed as DR. Once RDR deteriorates, the retina becomes vulnerable to damage, which then progresses in a self-amplifying cycle of oxidative stress, senescence, inflammation, vascular dysfunction, and pathological angiogenesis. The RDR concept offers a plausible explanation for the prolonged latency between the onset of DM and the manifestation of DR. RDR-based therapeutics, which enforce resilience to hyperglycemia-induced damage, will complement the current prophylactic strategy of reducing the level of blood glucose.

## Figures and Tables

**Table 1:** **Impact of senescence on a given stage of DR pathogenesis.** The hallmarks of early DR, the stage at which vascular dysfunction does not compromise vision, include microaneurysms, intraretinal hemorrhages, exudates, vascular tortuosity and cotton-wool spots. Advanced, vision-compromising hallmarks of DR include the following additional features: macular edema and neovascularization.

*Stage of DR Pathogenesis*	*Impact of Senescence*
Deterioration of RDR	Unknown
Vascular dysfunction that does not compromise vision	Exacerbates pathology by promoting SASP-mediated inflammation
Vision-compromising vascular dysfunction	Exacerbates or mitigates vascular dysfunction
